# Verses

**Published:** 1906-01

**Authors:** 


### VERSES.

### The Green Prevailed.

A green little boy in a green little way
 A green little apple devoured one day,
And the green little grasses now tenderly wave
 O'er the green little apple boy's green little grave.

---

### The Belated Mourners.

Dey wuz cryin' at his grave—
 Des a-moanin' ever'whare'!
Did dey ever cry fer 'im
 'Fo' he got down dar?

Dey wuz pilin' flowers on it—
 Some a-blazin' lak a star!
Did dey ever give 'im flowers
 'Fo' he got down dar?

Oh, dey done a heap er cryin'

 Hit wuz rainin' ever'whare'!

'Twuz de love dey should 'a' gin him

 'Fo' he got down dar!

*Frank L. Stanton.*

### LIFE.

"Oh, what is Life? 'Tis but a thought
 Sprung from the Eternal Mind,
And by the mighty Maker brought
 To us and all mankind.

"And like a thought it passes by
 Waits not for morn or even,
But heaves one longing, lingering sigh
 And flies again to heaven."

*Homer Judd—Brief.*

### PUNKIN YAM AN POSSUM.

Did yer ebber eet er Punkin Yam?
 Hits meat am red, an jucy too,
Sweet es shugger in any jam,
 No? Den deres er treat erwaitin' you.

Longside er possum een de dish,
 Er swimmin' een de gravy
Hit's de bess ob eetin' I kin wish
 Ur mer name aint Julius Davy.

Den agin fer er sorter 'sert,
 Ef deres room ter breve er sigh,
Hit aint gwine ter harm en hurt,
 Fer hit meks de bess ob tater pie.

*B. H. Teague.*

